# Is chronic obstructive pulmonary disease a risk factor for epistaxis after coronary artery bypass graft surgery?

**DOI:** 10.5830/CVJA-2014-061

**Published:** 2014

**Authors:** Faruk Cingoz, Bilgehan Savas Oz, Gokhan Arslan, Adem Guler, Mehmet Ali Sahin, Celalettin Gunay, Mehmet Arslan

**Affiliations:** Department of Cardiovascular Surgery, Gulhane Military Medical Academy, Etlik, Ankara, Turkey; Department of Cardiovascular Surgery, Gulhane Military Medical Academy, Etlik, Ankara, Turkey; Department of Cardiovascular Surgery, Gulhane Military Medical Academy, Etlik, Ankara, Turkey; Department of Cardiovascular Surgery, Gulhane Military Medical Academy, Etlik, Ankara, Turkey; Department of Cardiovascular Surgery, Gulhane Military Medical Academy, Etlik, Ankara, Turkey; Department of Cardiovascular Surgery, Gulhane Military Medical Academy, Etlik, Ankara, Turkey; Department of Cardiovascular Surgery, Gulhane Military Medical Academy, Etlik, Ankara, Turkey

**Keywords:** epistaxis, chronic obstructive pulmonary disease, coronary artery bypass surgery

## Abstract

**Background:**

Chronic obstructive pulmonary disease (COPD) has customarily been associated with increased surgical morbidity and mortality rates after coronary artery bypass graft surgery (CABG). The aim of this study was to determine whether there is a relationship between epistaxis and COPD after CABG surgery.

**Methods:**

There were 3 443 patients who consecutively underwent isolated CABG from January 2002 to March 2012. We retrospectively analysed the data of 27 patients (0.8%) with newly developed and serious spontaneous epistaxis, which required consultation with the Ear Nose and Throat (ENT) Department. The patients were divided into three groups according to severity of nasal bleeding. Twenty-one (77.7%) patients in the three groups had COPD.

**Results:**

There were 19 males (70%) and eight females (30%). Their ages ranged between 52 and 72 years (mean 61 ± 5). Fifty-five per cent of the patients had hypertension and 78% had COPD. The overall duration of hospital stay was six to 11 days (mean 7.9 ± 1.1). Epistaxis was seen particularly on the fourth and seventh days postoperatively and 17 patients (63%) were treated with anterior, posterior, or anterior and posterior nasal packing (group 1). Nasal bleeding was controlled with electrocautery in six patients (22%) (group 2), and four (15%) were treated with surgical excision and blood transfusions (group 3). All patients (100%) had a good recovery with no mortality.

**Conclusion:**

The high coincidence between epistaxis and COPD made us wonder whether COPD may be a risk factor for epistaxis after CABG surgery. However, we could not find any direct causative link between COPD and epistaxis in patients who had undergone CABG. Epistaxis was more common in patients with COPD and it was more serious clinically in patients who had both COPD and hypertension.

## Abstract

Epistaxis is the most common otolaryngological emergency that affects up to 60% of the population in their lifetime. Six per cent of all epistaxis cases require medical attention.^1^

Chronic obstructive pulmonary disease (COPD) is often considered a risk factor for postoperative morbidity and mortality after coronary artery bypass graft (CABG) surgery. Postoperative complications such as respiratory failure, re-intubation, sternal dehiscence, prolonged mechanical ventilation, rhythm disturbances and prolonged hospital stays are known complications of COPD in CABG patients.^2^

Epistaxis is a rare complication that is not directly related to heart surgery.^3^ Many factors affect bleeding after cardiac surgery, such as thrombolytic agents, hypertension, trauma and nasal oxygen therapy. Data on the association between epistaxis and CABG surgery is less clear.^4^ There is a paucity of published data regarding the management of epistaxis in patients with COPD who undergo CABG. We conducted this study to determine whether there was a relationship between epistaxis and COPD after CABG surgery.

## Methods

This was a retrospective study. All patients of any age who consulted at the Ear Nose and Throat (ENT) Department with a diagnosis of serious spontaneous epistaxis requiring at least one nasal pack after CABG surgery were included in the study. Patients were divided into three groups according to the severity of nasal bleeding, which was determined by treatment modality. The three procedures included packing (anterior, posterior or anterior-posterior) (group 1), treatment with electrocautery (under direct vision or via endoscopic guidance) (group 2) and surgical ligation of bleeding vessels (group 3).

The 3 443 patients who underwent isolated CABG from 2002 to 2012 were assessed in this study and follow up was obtained from a review of their charts. We focused on only objective data obtained from the medical records, and analysed a total of 27 (0.8%) patients with complete data who consulted at the ENT Department with a diagnosis of spontaneous and incipient epistaxis (Tables 1–3).

All patients were operated on via a median sternotomy, with standard cardiopulmonary bypass procedure and moderate hypothermia. Myocardial preservation was accomplished with intermittent antegrade delivery of St Thomas II solution. Cardiopulmonary bypass was initiated after anticoagulation with bovine lung heparin. The heparin was reversed by protamine at the end of the cardiopulmonary bypass. Nasal wet oxygen therapy (2–4 l/min) was initiated in all patients after extubation. Aspirin treatment (300 mg/day) was started after mediastinal bleeding had ceased in all patients. Hypertension was diagnosed if the systolic blood pressure (BP) was above 140 mmHg or diastolic BP was above 90 mmHg on two or more occasions.

Postoperative records up to discharge from hospital and available results related to the end-points were compared. The primary end-point was spontaneous epistaxis (with ENT consultation), requiring at least one nasal packing after CABG surgery, and any complications related to nosebleed and treatment, excluding death. The secondary end-point was death related to epistaxis.

## Statistical analysis

All available data were analysed with the computer program SPSS (Statistical Package for Social Sciences) for Windows 17.0 (Chicago, IL, USA). Descriptive statistical methods (number, percentage, mean, standard deviation) were used. Differences in variables were analysed using the Mann–Whitney U, Kruskal–Wallis and chi-square tests as appropriate, and p-values of less than 0.05 were considered significant.

## Results

A total of 27 patients with epistaxis consulted at the ENT Department. One patient had a history of epistaxis pre-operatively. There were 19 males (70 %) and eight females (30%), and their ages ranged between 52 and 72 years (mean 61 ± 5); 55% had hypertension, 78% COPD, 48% diabetes mellitus and 63% a history of smoking.

The overall duration of hospital stay ranged from six to 11 days (mean 7.9 ± 1.1) (Tables 1, 2, 3). Epistaxis was seen specifically on the fourth and seventh days postoperatively. Two patients had two or more epistaxis episodes within 15 days of discharge from hospital. According to the degree of bleeding, group 1 patients (*n* = 17, 63%) were treated with anterior, posterior, or anterior and posterior nasal packing. Group 2 patients (*n* = 6, 22%) were treated by electrocautery. Group 3 patients (*n* = 4, 15%) were treated by surgical ligation of the bleeding vessels and blood transfusions. These four patients in group 3 had two risk factors: hypertension and COPD (Table 2).

**Table 1:** Pre-operative patient characteristics

Male/female	19/8
Age (years), n (mean)	52–72 (61 ± 5.09)
Body mass index (kg/m2)	27.55 ± 1.36
Smoking, n (%)	17/27 (63)
Diabetes mellitus, n (%)	13/27 (48.1)
Hypertension, n (%)	15/27 (55.6)
aPTT (s)	31 ± 2.801
INR	1.107 ± 0.1107
Platelet count (/μl)	274444.44 ± 74644.456
LVEF (%)	55.05 ± 3.79

aPTT: activated partial thromboplastin time, INR: international normalised ratio, LVEF: left ventricular ejection fraction.

**Table 2:** Demographics and patient data

	*Total number (%)*		*Group 1*		*Group 2*		*Group 3*			
			*n*	*%*	*n*	*%*	*n*	*%*	*p*	*χ^2^*
Gender	27	Female	5	62.5	2	25.0	1	12.5	0.960	0.081
		Male	12	63.2	4	21.1	3	15.8		
Smoking	17 (62.9)	No	6	60.0	3	30.0	1	10.0	0.704	0.703
		Yes	11	64.7	3	17.6	3	17.6		
Diabetes mellitus	13 (48.1)	No	8	57.1	4	28.6	2	14.3	0.708	0.689
		Yes	9	69.2	2	15.4	2	15.4		
Hypertension	15 (55.5)	No	9	75.0	3	25.0	0	0.0	0.152	3.772
		Yes	8	53.3	3	20.0	4	26.7		
COPD	21 (77.7)	No	5	83.3	1	16.7	0	0.0	0.415	1.758
		Yes	12	57.1	5	23.8	4	19.0		

*Statistical significance was established as *p* < 0 .05. COPD: chronic obstructive pulmonary disease.

**Table 3:** Patient characteristics and haematological parameters in the three groups

*Variables*	*Group*	*n*	*Mean ± SD*	*KW*
Age	Group 1	17	61.760 ± 5.391	0.696
	Group 2	6	60.170 ± 4.708	
	Group 3	4	59.500 ± 5.000	
Hospital stay (days)	Group 1	17	7.470 ± 0.717	11.469
	Group 2	6	8.170 ± 1.169	
	Group 3	4	9.750 ± 0.957	
BSA (kg/m2)	Group 1	17	27.235 ± 1.427	2.454
	Group 2	6	28.033 ± 1.155	
	Group 3	4	28.200 ± 1.178	
LEVF (%)	Group 1	17	54.940 ± 3.944	0.400
	Group 2	6	56.170 ± 3.061	
	Group 3	4	55.750 ± 5.439	
Platelet count (per μl)	Group 1	17	252 352.940 ± 68 786.755	3.963
	Group 2	6	320 000.000 ± 56 213.877	
	Group 3	4	300 000.000 ± 100 000.000	
aPTT (s)	Group 1	17	30.530 ± 2.718	3.217
	Group 2	6	30.830 ± 3.251	
	Group 3	4	33.250 ± 1.708	
INR	Group 1	17	1.100 ± 0.117	0.371
	Group 2	6	1.133 ± 0.121	
	Group 3	4	1.100 ± 0.082	

*Statistical significance was established *p* < 0 .05 BSA: body surface area, LVEF: left ventricular ejection fraction, aPTT: activated partial thromboplastin time, INR: international normalised ratio.

All pre-operative patient characteristics and co-morbid factors between the groups were similar (*p* > 0.05) (Tables 1, 2, 3). Although all patients with epistaxis presented with discomfort in the postoperative period, all patients (100%) had a good recovery with no mortality. Group 3 patients had profuse nasal bleeding that needed surgical intervention, and both COPD and hypertension were diagnosed in all four of these patients.

## Discussion

Epistaxis is classified on the basis of the primary bleeding site being anterior or posterior. A common source of anterior epistaxis is the Kiesselbach plexus, an anastomotic network of vessels on the anterior portion of the nasal septum. Posterior bleeding occurs mainly from the branches of the sphenopalatine artery in the posterior nasal cavity or nasopharynx.^5^

There are many factors causing epistaxis, including environmental factors (humidity, temperature), local factors (inflammation, deviated septum and/or perforation, tumours, foreign bodies, aneurysm), systemic factors (hypertension, haematological abnormalities, renal failure, alcoholism, arteriosclerosis, telangiectasis), and medications affecting clotting (anticoagulants, non-steroidal anti-inflammatory drugs).^6^ The literature does not provide a precise definition on the severity of epistaxis, which is often based on subjective impressions (subjective evaluation of the volume of bleeding) or anatomical features, essentially posterior epistaxis.^7^

COPD is a known risk factor for morbidity and mortality in heart surgery. Postoperative complications such as respiratory failure, prolonged mechanical ventilation and oxygen uptake, re-intubation, sternal dehiscence, pulmonary infection, rhythm disturbances and prolonged hospital stays are known complications in COPD patients after CABG.^2^

We could not find any literature on epistaxis in patients with COPD undergoing CABG surgery. In COPD patients, drying and thinning of the nasal mucosa due to long-term nasal oxygen uptake or nebulised use of corticosteroids may cause epistaxis.^8^ Irritation by the endotracheal tube in the pulmonary system induces the cough reflex and coughing may cause sudden hypertension in the blood vessels in the nasal cavity. However we do not believe that in our cases, these factors were the cause of excessive nasal bleeding after CABG.

Hypertension and antiplatelet therapy may be a predisposing factor for nasal bleeding in COPD patients post CABG. Aspirin is thought to be a risk factor for epistaxis.^3^ The relationship between hypertension and epistaxis is unclear.^4^ In our study, neither hypertension nor aspirin were found to be independent risk factors for epistaxis.

However the presence of COPD in all patients (100%) with epistaxis, requiring surgical intervention and blood transfusion, induced us to conduct this study. Profuse nasal bleeding was seen if the patients had both COPD and hypertension. This analysis was conducted in our setting to identify the aetiological profile and to determine the outcome of treatment for epistaxis after CABG surgery. The results of this study may provide a basis for the planning of preventive strategies and the establishment of treatment guidelines.

There was profuse nasal bleeding requiring surgical intervention in all patients in group 3. Both COPD and hypertension were diagnosed in all four of these patients. Although there was no statistically meaningful results for nasal bleeding in these patients because of the low number of cases (*p* = 0.415), there was an interesting connection between COPD and hypertension after CABG surgery. Our results showed that both COPD and hypertension were present in patients with serious nasal bleeding after CABG surgery.

A limitation of this study was that the incidence of epistaxis in the early postoperative period after CABG was low. In our study, only 27 patients had epistaxis. These patients were divided into three groups according to the amount of nasal bleeding and the type of treatment, and each group contained only a few patients. There are also many causes of epistaxis after CABG.

## Conclusion

Epistaxis is a co-morbid factor in a small number of patients with COPD after CABG. It may result in a serious clinical situation with regard to the amount of nasal bleeding when seen in patients with COPD alone or with both COPD and hypertension. From to our results, we recommend that when COPD and hypertension coincide, cardiac surgeons should keep in their mind that serious nasal bleeding may occur in these patients after CABG surgery. If so, they should be sent immediately to an ENT specialist for appropriate treatment.
